# Effectiveness of pulmonary rehabilitation in patients with interstitial lung disease of different etiology: a multicenter prospective study

**DOI:** 10.1186/s12890-017-0476-5

**Published:** 2017-10-10

**Authors:** Roberto Tonelli, Elisabetta Cocconcelli, Barbara Lanini, Isabella Romagnoli, Fabio Florini, Ivana Castaniere, Dario Andrisani, Stefania Cerri, Fabrizio Luppi, Riccardo Fantini, Alessandro Marchioni, Bianca Beghè, Francesco Gigliotti, Enrico M. Clini

**Affiliations:** 1 Respiratory Diseases Unit-AOU Policlinico, Department of Medical and Surgical Sciences, University of Modena Reggio Emilia, Modena, Italy; 2 Respiratory Diseases Unit, Don Gnocchi IRCCS, Florence, Italy; 3 Rehabilitation Unit, Villa Pineta Hospital, Pavullo n/F, Modena, Italy; 4 Respiratory Diseases Unit, Cardiovascular Department, University of Padua, Padua, Italy

**Keywords:** Pulmonary rehabilitation, Interstitial lung diseases, Endurance test, Endurance time, Functional performance

## Abstract

**Background:**

Recent evidences show that Pulmonary Rehabilitation (PR) is effective in patients with Interstitial Lung Disease (ILD). It is still unclear whether disease severity and/or etiology might impact on the reported benefits. We designed this prospective study 1) to confirm the efficacy of rehabilitation in a population of patients with ILDs and 2) to investigate whether baseline exercise capacity, disease severity or ILD etiology might affect outcomes.

**Methods:**

Forty-one patients (IPF 63%, age 66.9 ± 11 ys) were enrolled in a standard PR course in two centers. Lung function, incremental and endurance cyclo-ergometry, Six Minutes Walking Distance (6MWD), chronic dyspnea (Medical Research Council scale-MRC) and quality of life (St. George Respiratory Questionnaire-SGRQ) were recorded before and at the end of PR to measure any pre-to-post change. Correlation coefficients between the baseline level of Diffuse Lung Capacity for Carbon monoxide (DLCO), Forced Vital Capacity (FVC), 6MWD, power developed during incremental endurance test, GAP index (in IPF patients only) and etiology (*IPF* or *non-IPF*) with the functional improvement at the 6MWDT (meters), at the incremental and endurance cyclo-ergometry (endurance time) and the HRQoL were assessed.

**Results:**

Out of the 41 patients, 97% (*n* = 40) completed the PR course. Exercise performance (both at peak load and submaximal effort), symptoms (iso-time dyspnea and leg fatigue), SGRQ and MRC significantly improved after PR (*p* < .001). Patients with lower baseline 6MWD showed greater improvement in 6MWD (Spearman r score = − .359, *p* = .034) and symptoms relief at SGRQ (*r* = −.315, *p* = .025) regardless of underlying disease.

**Conclusion:**

Present study confirms that comprehensive rehabilitation is feasible and effective in patients with ILD of different severity and etiology. The baseline submaximal exercise capacity inversely correlates with both functional and symptom gains in this heterogeneous population.

## Background

Interstitial lung diseases (ILDs) comprise a heterogeneous group of chronic conditions characterized by lung parenchymal involvement with different degrees of inflammation and fibrosis resulting in impaired gas exchange and restrictive physiology. Clinically, the development of irreversible and progressive parenchymal fibrosis leads to ventilatory constraint and abnormal lung mechanics with limited exercise capacity and dyspnea on exertion [1]. In particular, the impaired level of gas exchange seems to be the major cause leading to exercise intolerance in these patients [2]. Consequently, as ILD progresses, the patient’s daily activities decline early following symptoms (shortness of breath, tiredness, muscle fatigue). This reduction in everyday performance begins even before that ventilatory limitation with functional impairment occurs [3]. Furthermore ILD patients experience greater physical and social limitations once ventilatory constraint has established, reducing their functional reserves [2]. Finally, due to the progressive exercise limitation, individual’s health-related quality of life (HRQoL) is markedly affected [4].

Thus, the proper management of dyspnea represents a critical need for patients suffering from ILD. Pulmonary rehabilitation (PR) programs have been widely assessed and validated in patients with Chronic Obstructive Pulmonary Disease (COPD) [5], for which they have been proved to be effective in reducing respiratory and non-respiratory (i.e. peripheral muscle fatigue) symptoms and improving functional performance status which is consistent with improvement in HRQoL [6]. Nonetheless, there is a growing body of evidence showing that PR consisting of tailored and supervised training on aerobic and resistance exercises, breathing techniques and education sessions focused on self-management of symptoms and physical activity promotion could improve outcomes like dyspnea, functional capacity and quality of life also in patients with ILD [7–11]. However, the ultimate guidelines on diagnosis and management of Idiopathic Pulmonary Fibrosis (IPF) [1] provide a weak recommendation for PR in this particular form of ILD, due to the low quality of evidence on the real achievable gain. Furthermore, there is still no clear evidence regarding which type of ILD form (idiopathic VS non idiopathic) could benefit the most from a PR program.

With reference to this, Huppmann and colleagues prospectively investigated the effect of a 4-week PR program in a wide population of 402 patients with ILDs. The authors reported clinically relevant improvements in both functional exercise capacity and in HRQoL independent on the underlying disease (which were IPF, cryptogenic organizing pneumonia, hypersensitivity pneumonitis, sarcoidosis, or others) [12]. However, in a systematic review by Holland et al., patients with IPF seem to have lower improvement in functional capacity following PR as compared with others suffering from different etiologies of ILD [13].

Even more, the impact of disease severity on PR outcomes seems not to be well clarified in patients with ILDs. Lower lung derangement, less oxyhaemoglobin desaturation and lower level of secondary pulmonary hypertension have been associated with greater improvement in functional capacity in patients with IPF but were not predictors of benefit in those with other ILDs [14]. This might indicate that PR is likely to be effective in IPF at early stage of the disease, whereas different etiology of ILD may benefit regardless of disease severity. Indeed, in a prospective multicenter cohort study, Ryerson and colleagues had showed that patients with ILD (of whom around only one third were IPF) and a low functional capacity at baseline had greater benefit from rehabilitation [3].

In summary despite the beneficial effect of PR in ILD patients has been supported by a growing body of evidence, the ultimate IPF guidelines still provide a weak recommendation for PR [1] and 2) the impact of disease severity and ILD etiology on PR outcomes remains not well understood. The present prospective observational two-center study has been designed 1) to confirm the positive impact of pulmonary rehabilitation delivered both as in- and outpatient program in a population of patients with ILDs of different etiology [15] and 2) to further investigate whether baseline exercise capacity, disease severity or ILD etiology might differently affect clinical outcomes following a standard PR course.

## Methods

### Patient population and study design

Between January 2013 and January 2015, 41 consecutive patients with ILDs of different etiology were prospectively enrolled as in-patients (*n* = 30, 73%) or out-patients (*n* = 11, 27%) to perform a standard comprehensive PR program [6] both at “Villa Pineta” Rehabilitation Hospital in Pavullo n/F (Modena) and at “Don Gnocchi” Institute in Firenze. Patients not fulfilling criteria for PR [6] were excluded. Diagnosis of ILD was performed according to the ATS/ERS international consensus classification of idiopathic interstitial pneumonias [16].

The study had a prospective design. It was approved by the local review board and the Ethics Committee at both Institutions. All the individuals gave their informed consent to participate and to publish data on scientific journals.

**Fig. 1:**
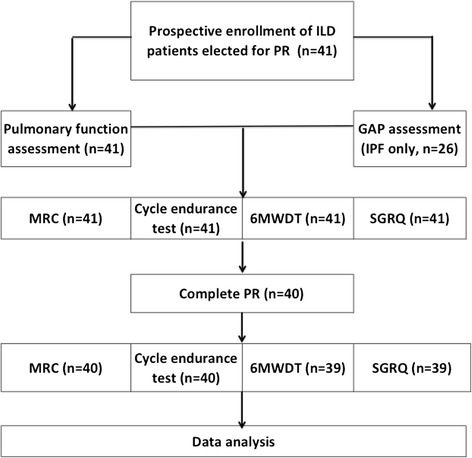
Study flowchart

The study flowchart is shown in Fig. 1.

### Assessment and outcomes

#### Functional status

Routine pulmonary function tests (forced vital capacity-FVC and forced expiratory volume in 1st second-FEV_1_) and diffusive lung capacity (DLCO), obtained with subjects in a comfortable seated position, were measured according to the American Thoracic Society/European Respiratory Society (ATS/ERS) recommendations [17]. Lung volumes were measured by volume-displacement body plethysmograph (Autobox DL 6200; SensorMedics, Yorba Linda, CA); the normal reference values were those by the European Community for Coal and Steel [18].

Blood gases were measured by an automated analyzer (IL-1650, Instrumentation Laboratory, Milan, Italy for the center of Modena and ABL 800, Radiometer, Copenaghen for the center of Florence) from an individual’s arterial sample taken when in resting condition while breathing room air.

Disease severity score of IPF patients was recorded using the GAP-staging system [19], which includes gender, age, forced vital capacity (FVC) and diffusion lung capacity for carbon monoxide (DLCO).

Incremental exercise testing (1-min increments of 10 W) to a symptom-limited maximum was performed on an electronically braked ergometer (Ergo-Metrics 800 s; SensorMedics) for lower limbs pre-post PR. The patients were familiarized with the apparatus days prior to the test; they were encouraged to keep exercising for as long as possible while pedaling at 50 to 60 rpm and were instructed to maintain work levels until they reached a symptom-limit. In addition, the six-minute walked distance (6MWD) was carried out according to the ATS/ERS guidelines [20]. All the subjects underwent two consecutive tests with the best result taken as representative.

A pulmonologist or a cardiologist and a rehabilitation therapist, unaware of the study purposes, closely supervised each exercise session; during the session, heart rate and peripheral oxygen saturation (SpO_2_) were monitored continuously.

#### Symptoms and health-related quality of life

The perceptions of dyspnea and limb efforts were determined at the beginning and at the end of 6MWD and every 2 min during cycle-ergometry using the modified Borg scale [21]. This scale is a vertical list with labeled categories describing increasing intensities of the perceived symptom (dyspnea or fatigue). Subjects were asked to rank the overall intensity of respiratory discomfort and leg effort by pointing to a score on a large scale from zero (none, i.e. no sensation at all) to 10 (maximum, i.e. the most severe sensation they had ever experienced).

Chronic exertional dyspnea was assessed by using the 5-point (0 to 4) MRC scale [22].

The St. George Respiratory Questionnaire (SGRQ) as validated into the Italian version was used to assess the patient’s health-related quality of life (HRQoL) [23]. Total and component (symptoms, activity and impacts) scores were recorded for analysis. This questionnaire was previously used also in patients with ILD [24, 25].

Maximal load and level of symptom achieved during incremental exercise test, endurance time and dyspnea recorded at iso-time during endurance test, distance walked, chronic dyspnea and HRQoL were used as outcome measures, suitable for a pre-to-post comparison.

### Pulmonary rehabilitation program

The two centers involved provided a standardized similar and shared PR course as recommended [6, 26]. PR program consisted of: 6-h/week individually exercise training, including endurance training for upper and lower limbs, 2 session of breathing techniques lasting 30 min for four to five times per week and 3 sessions of group education per week. Exercise training consist of aerobic (treadmill, stationary bikes) and resistance training (light weights, resistance bands) and included supervised cycle and supported arm ergometry, and leisure walking used as outcome measures, suitable for a pre-to-post comparison.

Breathing training consisted of breathing techniques (controlled and diaphragmatic breathing), pacing and energy conservation. Supplemental oxygen was delivered to maintain normal level of oxygen saturation. PR was tailored on the patient’s functional status and performance. The educational topics included medication and oxygen use, nutrition, panic control and relaxation techniques, as well as psychosocial support and issues of palliation and/or end-of-life related to the disease progression [6]. If needed, patients received psychosocial support. The PR program lasted at least 24 sessions of rehabilitation training and was conducted 6 days a week, once daily the first week and twice daily thereafter. Each rehabilitation session lasted at least 3 h.

### Statistical analyses

Per-protocol analysis was conducted by means of STATA 11.2 statistical package (StataCorp, Texas, USA). Data were obtained from prospectively maintained medical records and computerized databases. A *p* value lower than 0.05 was required for significance.

We used Kolmogorov–Smirnov test in order to assess the distribution of data showing that all data follow a normal distribution. Pre to post comparisons were performed by paired and unpaired *t*-test, χ square test and Wilcoxon rank sum test. Changes from baseline in 6MWDT (meters) and in Endurance Time (minutes) and SGRQ (total score) following PR were considered as indicators of improvement in functional capacity and HRQoL respectively [3, 27]. The statistical power expected for the present study population was calculated with reference to the primary outcomes and the minimum difference appreciable by our sample was assessed at ≥3% of baseline values for major outcomes.

With reference to etiology patient were divided in “IPF” and “non IPF”. Baseline DLCO, FVC and GAP index (for IPF patients only) were considered indicators of lung derangement and disease severity. The distance covered at 6MWD and the power (watt) developed during the incremental endurance test at baseline were taken to assess functional performance.

To asses correlation between baseline exercise capacity, disease severity and ILD etiology with functional and symptoms improvement a bivariate analysis by Spearman rank correlation coefficient was applied.

Results will be described as mean ± standard deviation (SD), except otherwise indicated.

## Results

Forty out of 41 patients referred to PR completed the program; one subject with hypersensitivity pneumonitis (non IPF) dropped out as he developed bacterial pneumonia in hospital, once he had completed 20 rehabilitation sessions. Among patients who completed the program the number of rehabilitation sessions performed ranged from 26 to 32 and did not differ between in and outpatients (*p* = .15).

Demographics and baseline data on function, symptoms, disease etiology and severity are shown in Table 1. Baseline features were comparable with no statistically significant difference between patients enrolled in the two centers and involved in the in- and outpatient program. Mean DLCO and volumes (overall mean FVC 74.5 ± 21.42% of predicted value) indicated a mild-to-moderate lung restriction. Notably, the overall mean GAP index score in IPF patients was 3.74 ± 1.69 over a maximum of 7. Twelve subjects presented abnormal gas exchange on effort and were prescribed for using oxygen as a supportive therapy. At baseline, the overall mean distance covered at the 6MWDT was 376.8 m ± 94.6 m and the overall mean endurance power developed during incremental exercise test was 57.5 w ± 23.7 w, which revealed a substantial reduction of exercise capacity both at submaximal and maximal performance test. Among patients with ILD other than IPF, 8 had pulmonary fibrosis associated to collagen vascular disease, 4 had chronic hypersensitivity pneumonitis, 2 had sarcoidosis and 1 had asbestos-related ILD.

**Table 1:** Baseline features of study population

Baseline characteristics	Overall	Center of provenience		*p*	Setting of PR program		*p*
		A^a^ (n. 20)	B^b^ (n. 21)		In (n. 30)	Out (n. 11)	
Age (years)	66.9 ± 10.9	67.8 ± 9.7	65.4 ± 11.1	.74	70.3 ± 15.7	62.4 ± 7.1	0.12
Gender (males: females)	27: 14	13: 7	14: 7	.99	21: 9	8: 3	0.99
BMI (kg/m^2^)	28.1 ± 3.4	27.8 ± 2.4	28.3 ± 1.1	.84	27.6 ± 7.4	29.3 ± 1	0.46
Diagnosis (IPF: non IPF)	26: 15	13: 7	13: 8	.99	18: 12	8: 3	0.72
Smoking history (yes: no)	30: 11	16: 4	14: 7	.48	21: 9	9: 2	0.69
GAP index -for IPF only-	3.74 ± 1.69	4.14 ± 2.19	3.24 ± 1.15	.11	4.36 ± 3.19	3.24 ± 1	0.345
Inpatients (in: out)	30: 11	15: 5	15: 6	.99	–	–	–
O_2_ therapy (yes: no)	12: 29	5: 15	7: 14	.73	10: 20	2: 9	.46
O_2_ therapy (continuous: during effort)	8: 4	3: 1	5: 3	.99	7: 3	1: 1	.99
Arterial pO_2_ (mmHg)	68 ± 12	70 ± 10	66 ± 7	.14	64 ± 14	71 ± 3	.11
Arterial pCO_2_ (mmHg)	37 ± 4	38 ± 5	37 ± 2	.4	37 ± 4	37 ± 1	.99
FVC (% predicted)	74.5 ± 21.4	72.4 ± 16.4	75.8 ± 7.4	.39	70.4 ± 16.4	77.8 ± 8.4	.16
FEV_1_ (% predicted)	78.7 ± 20.7	79.6 ± 19.6	77.4 ± 21.7	.74	72.6 ± 19.6	79.4 ± 12.7	.29
DLCO (% predicted)	45.5 ± 20.9	43.3 ± 21.2	46.9 ± 19.5	.57	42.4 ± 19.2	47.9 ± 12.5	.38

^a^Center A = “Don Gnocchi” Institute, Firenze, Italy

^b^Center B = “Villa Pineta” Rehabilitation Hospital in Pavullo n/F, Modena, Italy. Continuous variables are indicated as mean ± standard deviation, non continuous as n

**Fig. 2:**
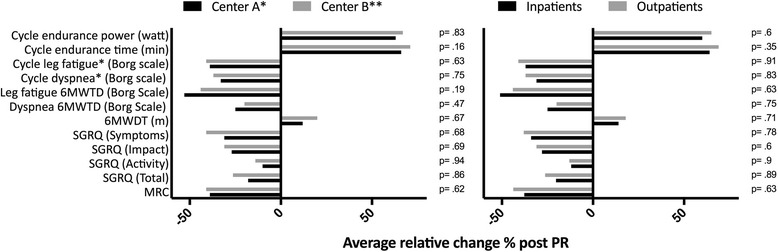
Effect of PR setting and center of enrollment on PR effectiveness expressed in terms of relative change from baseline. Cycle dyspnea and leg fatigue were assessed at isotime. * Center A = “Don Gnocchi” Institute, Firenze, Italy. ** Center B = “Villa Pineta” Rehabilitation Hospital in Pavullo n/F, Modena, Italy

**Fig. 3:**
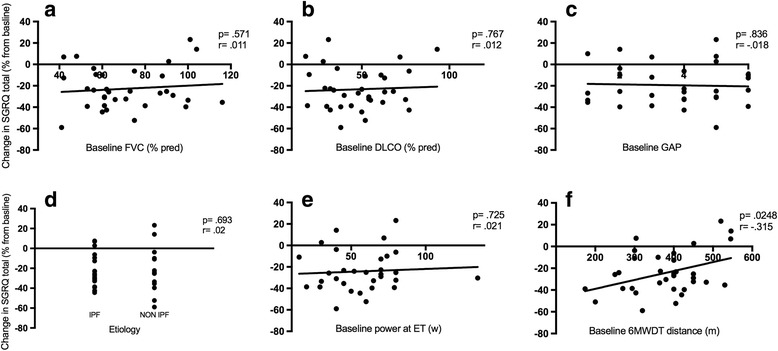
Correlation between baseline FVC (panel **a**), DLCO (panel **b**), GAP index (panel **c**), ILD etiology (panel **d**), power developed at endurance test (ET) (panel **e**), distance covered at 6MWDT panel **f**)  and change in 6MWDT distance (%) after PR. Statistical significant is indicated by *p* value while correlation is indicated by the Pearson’s correlation coefficient r

**Fig. 4:**
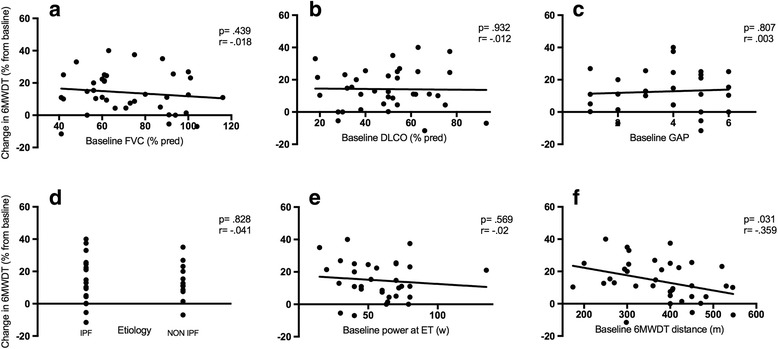
Correlation between baseline FVC (panel **a**), DLCO (panel **b**), GAP index (panel **c**), ILD etiology (panel **d**), power developed at endurance test (ET) (panel **e**), distance covered at 6MWDT panel **f**) and change in SGRQ (total) after PR. Statistical significant is indicated by *p* value while correlation is indicated by the Pearson’s correlation coefficient r

**Fig. 5:**
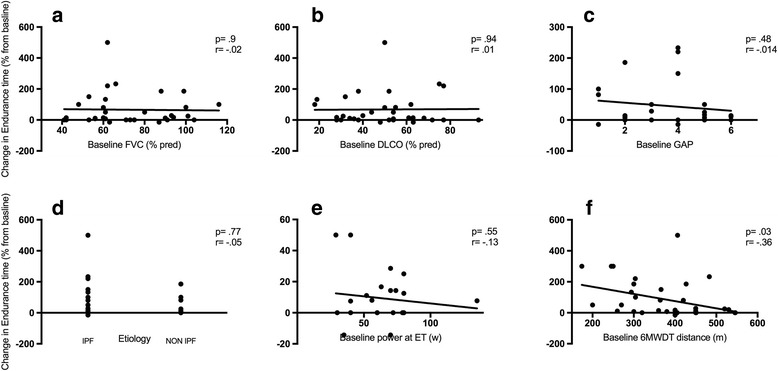
Correlation between baseline FVC (panel **a**), DLCO (panel **b**), GAP index (panel **c**), ILD etiology (panel **d**), power developed at endurance test (ET) (panel **e**), distance covered at 6MWDT panel **f**) and change in Endurance Time after PR. Statistical significant is indicated by *p* value while correlation is indicated by the Pearson’s correlation coefficient r

In Table 2 outcome measures and their absolute or relative change following PR are displayed: the standard rehabilitation course resulted in a significant improvement in all the pre specified outcomes, being not influenced by PR setting and center of enrollment (see Fig. 2). Distance covered, peak and endurance exercise performance increased after PR, while symptoms score at peak exercise or iso-time decreased substantially. Notably, SGRQ score improved in all the categories. The minimal clinically important difference (MCID) in 6MWD and SGRQ was achieved by 75.6% and 80.3% of patients respectively. Nor IPF diagnosis neither other baseline features significantly influenced the achievement of the MCID in the major outcomes. Lower 6MWD covered at baseline significantly correlated with improvement in 6MWD (Fig. 3, panel f), endurance time at cycle ergometer (Fig. 5, panel f) and in SGRQ (total score) (Fig. 4, panel f) following PR, whereas power reached at incremental endurance test, FVC, DLCO, GAP index score and etiology did not (see also in Figs. 3, 4 and 5 panels a to e).

**Table 2:** Outcome measures with absolute and relative change following PR

Outcomes	n	Before PR	Post PR	Absolute change	Relative change (%)	*p*
MRC	40	2.8 ± 0.8	1.7 ± 1.1	- 1.1 ± 0.8	−40.8 ± 35	<.001
SGRQ (Total)	39	50.6 ± 13.9	38.5 ± 13.7	- 12.1 ± 11.1	−23.3 ± 19	<.001
SGRQ (Activity)	39	67.6 ± 14	589 ± 19.6	- 8.8 ± 17.6	−12.5 ± 24.7	0.009
SGRQ (Impact)	39	43.7 ± 19.1	29.9 ± 15.9	- 13.7 ± 14.6	−29.9 ± 41.5	<.001
SGRQ (Symptoms)	39	40.4 ± 20.9	26.7 ± 20.8	- 17 ± 19.8	−36 ± 22.1	<.001
6MWDT (m)	39	376.8 ± 94.6	430.9 ± 96.4	54.1 ± 55.4	16.7 ± 37.8	<.001
Dyspnea 6MWTD (Borg Scale)	39	5.2 ± 2.3	3.8 ± 2.2	−1.4 ± 2.1	−23.4 ± 40.1	.015
Leg fatigue 6MWTD (Borg Scale)	39	3.5 ± 2.7	2 ± 2.2	−1.6 ± 1.5	−48.1 ± 71.5	.006
Cycle dyspnea^a^ (Borg scale)	40	6.1 ± 2	4.2 ± 2.7	- 1.8 ± 1.9	−33 ± 35.1	<.001
Cycle leg fatigue^a^ (Borg scale)	40	5.9 ± 2.1	3.7 ± 2.7	- 2.2 ± 2	−39.5 ± 106	<.001
Cycle endurance time (min)	40	7.7 ± 3.8	12.5 ± 8.4	4.8 ± 6.9	66 ± 108.1	<.001
Cycle endurance power (watt)	40	57.5 ± 23.7	88.2 ± 57.1	31 ± 53.5	63.4 ± 33.9	.003

Each outcome value is reported as mean value ± standard deviation (SD); *p* value are referred to relative changes

^a^Cycle dyspnea and leg fatigue were assessed at isotime

## Discussion

In this study we evaluated the impact of a standard comprehensive PR program conducted in the real life in patients with ILD of different etiology, functional performance and lung impairment. Overall, findings confirm that these patients are likely to gain large benefits from PR delivered as in and outpatient setting, both in terms of exercise performance, symptoms, and perceived quality of life regardless of underlying disease. In particular, our study extends the concept that patients with both IPF and non-IPF showing lower submaximal exercise capacity at baseline are more likely to benefit in terms of exercise performance and HRQoL.

Multiple aspects deserve discussion.

First, the extent of improvements experienced by the enrolled ILD-patients seems even greater than that reported in previous studies. In particular, the absolute change in the average distance covered at the 6MWDT after PR has been 54.1 ± 55.4 m (+ 16.7% from baseline, *p* < .001), compared to the 44.34 m (95% CI 26.04 to 66.64) as reported in a recent meta-analysis [28, 29]; furthermore the average reduction seen in the MRC score for dyspnea has been greater than 1 (− 1.1 ± 0.8, *p* < .001). The observed major benefits might be due to the peculiar baseline features of the study population, constituted of ILD patients with mild to moderate impairment of respiratory function and a measurable exercise capacity (although substantially reduced as expected) even at peak intensity. The permissive level of lung function derangement let almost all the patients to complete the rehabilitation program (only 1 patient left the study protocol for the onset of infectious pneumonia), with increased exercise tolerance both at submaximal and maximal performance. Thus we can postulate that less deranged lung in interstitial diseases may provide greater chance to successfully undertake and complete rehabilitation training, issuing its positive results over a limited baseline functional capacity.

Second, data from our study also show how ILD patients of different nature and severity could equally gain benefits from PR without any significant difference in terms of improvement. In particular the nature of the fibrotic lung changes does not impact the positive effect of PR. As a matter of fact, regardless of the etiology, patients affected with ILD share common clinical features with distressing dyspnea, exercise intolerance and persistent cough [3]. What might differentiate IPF from other forms of ILD, especially non-idiopathic, is the systemic involvement of the underlying disease that could affect the adherence to exercise training, thus reducing the effectiveness [10]. Therefore, our study has demonstrated that PR could provide benefits irrespectively of the nature of ILD and the ensuing eventual systemic involvement. Researches have widely questioned the impact of disease severity and the subsequent functional limitation on the effect of PR in ILD patients [8]. Ryerson and colleagues showed how a lower baseline 6MWD could predict larger improvement in distance covered after PR [3]. Similarly, those with relatively higher levels of baseline dyspnea experienced major prolonged symptoms relief [14]. Conversely, other studies show that greater and sustained improvements in functional capacity following exercise training have been described when patients present a higher baseline FVC and less desaturation on the baseline 6MWDT [30], suggesting that PR could be more effective if offered earlier in the disease course. This discordance may be explained by differences in the patient population (e.g. the presence of comorbidities) or study design (cohort vs randomized trial). In our study the different level of lung derangement expressed by levels of FVC and DLCO do not affect PR benefits. This suggests that motivated ILD patients with functional impairment from mild to moderate should be early referred to PR because they are likely to benefit.

Third, our study confirms that patients with lower baseline functional performance may experience the greater clinical benefit in terms of exercise capacity and symptoms relief. These data confirmed what already reported by Ryerson and colleagues [3], adding a significant correlation with lower submaximal baseline functional capacity and greater HRQoL improvement after PR. It seems interesting to stress that the power reached at incremental endurance test performed at baseline did not inversely correlate with functional improvement after PR. In a relatively recent study, Holland and colleagues directly compared the cardiorespiratory responses during 6MWDT and cardiopulmonary exercise test (CPET) in patients with ILD [31]. They showed how desaturation on exertion was higher during 6MWDT than during CPET independently on the physiological load. This seems due to the increased alveolar ventilation reached during cycling, with a subsequent offset of oxyhaemoglobin desaturation through augmentation in partial pressure of oxygen in arterial blood (PaO_2_). They conclude that 6MWDT, eliciting high but submaximal functional responses in ILD patients, is a unique tool to derive information across the range of disease severity. Our study enhances this concept suggesting that 6MWDT seems even more sensitive than incremental endurance test in identifying ILD patients with greater functional improving potential. It is worth to note that patients with a higher baseline 6MWD showed less increase in distance covered after PR, probably due to a reduced capacity for further improvement. Moreover a greater 6MWDT was not correlated with lack of improvement in other functional or symptomatic outcomes, thus confirming that physical intervention (rehabilitation) is a critical approach even in patients with a higher level of baseline exercise capacity.

Our study presents several limitations; first of all this was a preliminary trial whose explorative nature resulted in the lack of a sample size computation analysis. A subsequent study with a sample size computed on the basis of the evidence provided by the present study is needed. Secondly the limited size of the study population did not allow detailed subgroup analyses. In addition, the lack of a control group could limit the likely significance of our findings, while the lack of follow-up after PR did not allow to assess the persistence of the reported benefits over time nor comparing it between diagnosis (e.g. IPF vs non-IPF). Furthermore, all the patients enrolled showed a baseline mild to moderate impairment of lung function, without any case of severe lung derangement. This did not let to evaluate the effect of PR model on the more compromised patients and to investigate whether a more severe functional limitation could significantly affect the improvement after PR.

Future research is therefore required to better define the subset of ILD patients that can benefit most from exercise training and investigate whether different and/or more specific modalities should be used to enhance gains following PR in patients with more severe disease.

## Conclusions

There is a growing body of evidence to support pulmonary rehabilitation as a fundamental treatment for patients with lung fibrosis that should be early referred to tailored exercise programs. Our study has demonstrated that in ILD patients the level of lung derangement or etiology at baseline do not affect outcomes improvement following rehabilitation. Moreover, a lower derangement in lung function may provide a greater chance to successfully undertake and complete PR. Since the degree of improvement across measured outcomes is remarkable, this suggests that pulmonary rehabilitation should be a first line therapy for managing symptomatic patients with ILD of different nature and mild to moderate severity.

## Abbreviations

6MWD: Six Minutes Walking Distance
ATS: American Thoracic Society
CPET: Cardiopulmonary exercise test
DLCO: Diffuse Lung Capacity for Carbon monoxide
ERS: European Respiratory Society
ET: Endurance test
FVC: Forced vital capacity
GAP: Gender age pulmonary function
HRQoL: Health related quality of life
ILD: Interstitial lung disease
IPF: Idiopathic Pulmonary Fibrosis
MCID: Minimal clinically important difference
MRC: Medical Research Council
PaO_2_: Partial pressure of oxygen in arterial blood
PR: Pulmonary rehabilitation
SGRQ: St. George Respiratory Questionnaire
